# Evaluation of Filamentous Fungi and Yeasts for the Biodegradation of Sugarcane Distillery Wastewater

**DOI:** 10.3390/microorganisms8101588

**Published:** 2020-10-15

**Authors:** Graziella Chuppa-Tostain, Melissa Tan, Laetitia Adelard, Alain Shum-Cheong-Sing, Jean-Marie François, Yanis Caro, Thomas Petit

**Affiliations:** 1 Competitiveness Cluster Qualitropic, 5 rue André Lardy, 97438 Sainte-Marie, Réunion Island, France; graziella.tostain@qualitropic.fr; 2 Laboratoire de Chimie et Biotechnologies des Produits Naturels, Université de la Réunion, CHEMBIOPRO (EA 2212), 15 Avenue René Cassin, 97490 Sainte Clotilde, Réunion Island, France; melissa.tan@univ-reunion.fr (M.T.); alain.shum@univ-reunion.fr (A.S.-C.-S.); yanis.caro@univ-reunion.fr (Y.C.); 3 Département HSE, IUT de la Réunion, 40 Avenue de Soweto Terre-Sainte, BP 373, 97455 Saint-Pierre CEDEX, Réunion Island, France; 4 Laboratoire de Physique et Ingénierie Mathématique pour l’Energie et l’EnvironnemeNT (PIMENT), Université de la Réunion, 117 rue Général Ailleret, 97430 Le Tampon, Réunion Island, France; laetitia.adelard@univ-reunion.fr; 5 Laboratoire d’Ingénierie des Systèmes Biologiques et des Procédés, INSA de Toulouse, UMR INSA/CNRS 5504—UMR INSA/INRA 792, 135 Avenue de Rangueil, CEDEX 4, 31077 Toulouse, France; fran_jm@insa-toulouse.fr

**Keywords:** sugarcane, distillery waste water, molasses spent wash, vinasse, fungi, yeasts, bioremediation, COD, discoloration

## Abstract

Sugarcane Distillery Spent Wash (DSW) is among the most pollutant industrial effluents, generally characterized by high Chemical Oxygen Demand (COD), high mineral matters and acidic pH, causing strong environmental impacts. Bioremediation is considered to be a good and cheap alternative to DSW treatment. In this study, 37 strains of yeasts and filamentous fungi were performed to assess their potential to significantly reduce four parameters characterizing the organic load of vinasses (COD, pH, minerals and OD_475nm_). In all cases, a pH increase (until a final pH higher than 8.5, being an increase superior to 3.5 units, as compared to initial pH) and a COD and minerals removal could be observed, respectively (until 76.53% using *Aspergillus terreus* var. *africanus* and 77.57% using *Aspergillus niger*). Depending on the microorganism, the OD_475nm_ could decrease (generally when filamentous fungi were used) or increase (generally when yeasts were used). Among the strains tested, the species from *Aspergillus* and *Trametes* genus offered the best results in the depollution of DSW. Concomitant with the pollutant load removal, fungal biomass, with yields exceeding 20 g·L^−1^, was produced.

## 1. Introduction

In 2012, 83 billion liters of ethanol was produced worldwide, from which a third was from Brazil [1,2]. The European directive 2009/28/CE, relating to the promotion of the use of energy from renewable resources, also called RED (Renewable Energy Directive), sets, for each Member State, a binding target of a 10% share of renewable energy in the transport field in 2020. Biofuels production from waste, residues, non-food cellulosic material and lignocellulosic origin is particularly incited [3]. In Reunion Island, the sugarcane industry is one of the most important agricultural and economic activities of the French oversea department, and is located in the Indian Ocean [4].

In 2011, the three rum distilleries still active on the island produced 106,430 Hectoliters of Pure Alcohol (HPA) from sugarcane fermentation and molasses distillation [5]. The rum production is accompanied by the generation of a stillage called vinasse or distillery spent wash (DSW). Owing to the nature of the process, DSW are effluents with a high pollutant load, meaning high Chemical Oxygen Demand (COD, generally in the range of 100 to 150 g O_2_ L^−1^), a low pH (between 4.5 and 5.5) and a high optical density [6,7,8]. DSW also generally contains a high amount of potential nutrients such as nitrogen, phosphorous, potassium, sulfur and a large amount of micronutrients like calcium or magnesium [8]. Moreover, it is characterized by a dark brown color due to the presence of colored molecules such as phenolic acids, caramels from overheated sugars and furfurals from acid hydrolysis and melanoidins [9,10,11].

Melanoidins are dark brown polymers of low and high molecular weights that result from the Maillard reaction. This reaction, which happens at high temperatures and low pH, is a non-enzymatic browning reaction that results from condensation between reducing sugars and amino compounds [12], leading to molecules with complex structures [13]. It has been shown that less than 10% of the melanoidins present in the stillage can be degraded through conventional anaerobic–aerobic treatments [7,14]. Godshall (1999) showed that the amount of phenolic acids is higher in cane molasses stillage in comparison to beet molasses [15]. Depending on the sugarcane nature and the industrial processes used, intrinsic composition of DSW can vary significantly. Indeed, recent studies concerning COD show that vinasses from Indian and Mexican distilleries have COD around 104 and 121 g O_2_ L^−1^, respectively, whereas the COD of vinasses from Brazilian distilleries are two and three times less, i.e., 42 g O_2_ L^−1^. Moreover, proportions of potassium contained in DSW can range from 2.3 to 8.77 g·L^−1^ [16,17,18,19]. These characteristics make DSWs hazardous compounds when, for example, they are discharged in natural waterways. DSW can cause significant environmental problems by reducing the oxygenation of the water, causing eutrophication of contaminated waterways and creating toxic effects on aquatic organisms. Due to the presence of putrescible organics like skatole, indole and other sulfur compounds, DSW produces an obnoxious smell [20]. European and French regulations describe the strict specifications for industrial effluents. Their COD value must be less than 125 mg O_2_ L^−1^ [21]. 

Among the different ways of treatment, bioremediation offers a good perspective as it constitutes a cheaper and easier technique compared to physico-chemical technologies. Different types of microorganisms can be used for bioremediation, namely bacteria, microalgae, yeasts or filamentous fungi [7,8,22]. Due to their rapid growth, yeasts are widely used in DSW treatment. Among the 203 yeast strains tested, Akaki and collaborators showed that strains from *Hansenula, Debaryomyces* and *Rhodotorula* genus could remove a range of 32–38% of COD contained in a diluted and supplemented DSW medium [23]. Unlike yeasts, filamentous fungi have slower growth but their broad extracellular hydrolytic enzymes allow for the assimilation of complex carbohydrates without prior hydrolysis by another technique. Moreover, they are less sensitive to nutrients, aeration, temperature and pH variations [24]. In their study, Sirianuntapiboon and collaborators described the potential of 228 fungal strains to discolor molasses. Among them, nine strains, including four species from *Aspergillus* genus, a strain of *Trametes versicolor* and four other unidentified strains, showed discoloration yields above 50% [25,26]. 

A literature review indicated that species of filamentous fungi such as Penicillium, Aspergillus (A. niger, A. oryzae and A. terreus), Galactomyces geotrichum, Trametes versicolor, Phanerochaete chrysosporium and Flavodon flavus and the yeasts Candida tropicalis, P. jadinii and Issatchenkia orientalis had already been highlighted because of their ability to remove refractory compounds from distillery wastewater [7,8,20,27,28,29,30,31,32,33,34,35,36]. However, most of these strains were tested in different and heterogeneous conditions and not all on vinasse from sugarcane distilleries. In this study, we report a comprehensive and standardized screening program, including 37 strains of yeasts and filamentous fungi selected from the abovementioned literature and from our own selection (provided from laboratory fungal strain collections) for their ability to degrade complex compounds of DSW from sugarcane. The capacity of each strain to grow and metabolize the substrates contained in vinasse was evaluated by following the evolution of a number of physicochemical parameters such as pH, COD, mineral matter, optical density and microbial biomass production.

## 2. Materials and Methods

### 2.1. Biological Material and Growth Conditions

Sugarcane Distillery Spent Wash (DSW) was collected directly at the column outlet (between 85 °C and 100 °C) from “Rivière du Mât” sugarcane distillery, Saint-Benoit, Reunion Island. After cooling, DSW was stored in small sterile bags at −20 °C until used. The yeasts and filamentous fungi strains used in this study were purchased from BCCM (Brussels, Belgium) strain collections (Table 1). The 37 selected strains used in this study were chosen according to their specific properties to degrade complex molecules and particularly DSW. 

In order to be able to measure the depollution potential of the strains, independently of their capacity to grow on this medium, we decided to uncouple the depollution ability of the strain and the biomass production. Pre-cultures were therefore prepared by inoculating a full loop of 48 h growing cells on basal agar plate (PDA—Potatoes Dextrose Agar from Biotop) in sterile Malt Agar broth (MA—Merck, Germany) and incubated for 72 h. At the end of the incubation in MA broth, the total biomass formed was aseptically harvested by centrifugation, washed twice in sterile milliQ water and used for the inoculation of the main cultures. The main culture experiments contained 50 mL of autoclaved DSW (121 °C, 20 min). Yeast and filamentous fungi biomass harvested from pre-cultures were inoculated at a concentration of 10^5^ cells (yeasts) or spores (filamentous fungi) per mL in the main culture flasks and incubated under aerobic conditions at 110 rpm and 25 °C for 10 days. Biomass and culture broth were then separated by filtration (filamentous fungi) or centrifugation (yeasts). Filtrations were performed on a Buchner system using Whatman filter No. 1. All centrifugations were carried out for 15 min at 8500 rpm. 

### 2.2. Physico-Chemical Analysis

Cells harvested by centrifugation or filtration were incubated for 24 h at 105 °C for biomass determination. Supernatants and filtrates were stored at −20 °C before being used. The pH of broth medium during fermentation was measured using a pH-meter Denver Instrument (Germany). To evaluate the mineral content, ashes were measured by the incineration of 10 g of broth medium at 550 °C for 3 h in a muffle furnace Nabertherm Controller B170 (Lilienthal, Germany) [37]. COD measurements were carried out using Hanch Lange diagnostic kits (LCK 914) and measured spectrophotometrically with a DR 2800 spectrophotometer (Hach Lange, Dusseldorf, Germany). When necessary, the samples were adequately diluted with sterile deionized water and analyzed according to the manufacturer’s instructions. 

Absorbance of filtrates or supernatants was measured at 475 nm (corresponding to melanoidins) using a spectrophotometer Genesys 10 UV Scanning (Waltham, MA, USA) according to [19]. The discoloration yield was calculated according to the following equation:

(1) $$\mathrm{Discoloration}\;(\%)=\;\frac{I-F}{I},$$

with *I* = Initial absorbance (Control) and *F* = Absorbance after aerobic fermentation. All assays were performed in triplicates.

### 2.3. Chemometrics

Multivariate statistics, including Principal Component Analysis (PCA) and Hierarchical Cluster Analysis (HCA), were employed to investigate the relationships among species with similar performances concerning biomass production and variations of pH, mineral content, COD and OD_475nm_. PCA using Pearson correlation is a statistical method used in order to combine the original parameters (physicochemical variables) into several new uncorrelated components without losing significant information. The aim of this statistical method is to explain the variance–covariance structure of an experimental data set using a new set of coordinate systems. Every new principal component consisted of the linear combination of the original variables [38]. This method enabled us to define the characteristics of specific groups of strains. Hierarchical cluster analysis (HCA) was then used to identify the strains belonging to these groups. HCA is a statistical method to search for homogeneous clusters based on measured parameters. The hierarchical clustering process is represented by a dendrogram, in which each step of the clustering process is illustrated by a connection in the tree. Differences between these classes were tested with average Euclidean distances using the Ward method based on a variance approach. This Ward method provides a simple approach to approximate, for any given number of clusters, the partition minimizing the within-cluster inertia or “error sum of squares”. In this study, the method was performed with the aim of minimizing the sum of the squares of any two clusters that could be formed at each step. The clusters were then fused in order to reduce the variability within a cluster. Further, the fusion of two clusters resulted in a minimum increase of the “error sum of squares” [39]. These analyses were performed thanks to XLSTAT programs (Addinsoft, Inc., Paris, France). 

## 3. Results and Discussion

### 3.1. Effect of Aerobic Treatment on Chemical Oxygen Demand (COD)

The effect of the treatment of DSW on COD was found to be highly dependent of the strains used for aerobic treatment (Table 1). Aerobic fermentation of DSW by *Phanerochaete chrysosporium*, *Flavodon flavus*, *Fusarium proliferatum* and *Gibberella fujikuroi* appeared to be less efficient strains for COD reduction, with 23.5%, 28%, 34% and 38%, respectively, whereas *Aspergillus terreus var africanus*, *A. parasiticus*, *Trametes hirsuta*, *T. versicolor* and *A. terreus var. terreus* showed the highest decrease in COD (76.53%, 74.60%, 74.01%, 73.64% and 73.5%, respectively). Notably, the 9 *Aspergillus* and anamorph strains used in this study were among the most effective strains for COD reduction (COD reduction was higher than 65% for all 8 *Aspergillus* strains and 58.65% for *Fennellia flavipes*), indicating that these strains are particularly interesting for their reduction of the pollution load of DSW. COD reduction by *Pichia jadinii* and *Penicillium* sp. could reach 40.91% and circa 62%, respectively.

Some of our results were consistent with other published works. For instance, Gonzalez et al. (2000) reported a high COD reduction (62%) on diluted molasses spent wash treated by *Trametes* spp. [14]. Benito et al. (1997) also found that *T. versicolor* was able to reduce COD by more than 70% on supplemented sugar beet molasses [40]. Similarly, a reduction of 46% and 65% of COD was found for *P. jadinii* and *Penicillium* sp., respectively [30,41]. Aerobic treatment of cane molasses stillage with *A. niger* and *A. oryzae* led to a COD reduction of up to 78% and 88%, respectively [31,32,33]. On the contrary, Garcia et al. (1997) found that *A. terreus* lowered the COD of DSW by only 29% [34]. Surprisingly, our results using *P. chrysosporium* and *F. flavus* were found to be well below the observed values from the literature with, respectively, a 73% COD reduction on DSW supplemented with yeast extract and 80% on diluted DSW [42,43]. This difference may be explained by the fact that these studies were carried out on diluted and supplemented DSW, while we used crude DSW in our study. Moreover, COD reduction is generally concomitant with the discoloration of the vinasse. In their study, Fahy and collaborators (1997) showed that a sugar addition in the medium could significantly improve the depollution rate of vinasse by *P. chrysosporium* [44]. From these results, the efficiency of the strains to reduce COD is strongly dependent of the origin of the vinasse used (beet or cane for example) and their complementation with other sources of nutriments.

### 3.2. Effect of Aerobic Treatment on Colour

The effect of aerobic treatment of DSW on color was studied using optical density of DSW supernatant at 475 nm [19]. Consistent with the COD reduction, we found that the strains showing the highest reduction of color belong to *Aspergillus* and *Trametes* genus (Table 1). For instance, *Aspergillus parasiticus*, *A. alucateus*, *A. terreus var. terreus* and *A. itaconicus* led to a decrease of OD_475 nm_ up to 42.46%, 41.88%, 38.84% and 35.36%, respectively (Table 1). Similarly *Trametes hirsuta* and *T. versicolor* reached up to 42.46 and 32.46% of decolourisation of DSW. DSW treatment with *A. flavus, A. niger*, *A. oryzae*, *Fenellia flavipes*, *Flavodon flavus* and *Phanerochaete chrysosporium* also led to decolourization of DSW but to a lesser extent (OD_475 nm_ reduction was comprised of between 20 and 27%). Surprisingly, we observed that aerobic treatment of DSW by the yeasts (belonging to *Candida*, *Clavispora*, *Cryptococcus*, *Galactomyces*, *Issatchenkia*, *Komagatella*, *Pichia* and *Saccharomyces* genus) and by *Fusarium sporotrichoides* and *Penicillium verrucosum* resulted in small to high increase of OD_475 nm_. The most important increases of colourization were obtained for *Saccharomyces cerevisiae* (90.91%), *P. verrucosum* (68.99%), *C. dubliniensis* (52.53%), *P. jadinii* (41.08%), *F. sporotrichoides* (40%), *Issatchenkia orientalis* (39.73%), *C. tropicalis* (38.72%), *P. guilliermondii* (38.38%), *Pseudozyma antarctica* (36.03%), *Cryptococcus albidus* (35.35%) and *C. glabrata* (30.64%). The other yeasts species (*C. albicans*, *P. angusta*, *Komagatella pastoris*, *Galactomyces geotrichum*, *Rhizopus microsporus. var oligosporus* and *Thanatephorus cucumeris*) showed only limited colorization of the broth (less than 18%). A study has already noticed the increase of color after treatment. Kumar and collaborators (1998) reported that the optimum discoloration was closely related to the optimal growth and that the overall discoloration was obtained in the pH range of between five and eight, whereas at extreme pH levels, an increase in color was observed [42]. We can then hypothesize that the coloration observed in this study is probably due to the high final pH reached at the end of the process (Table 1). Finally, Kumar and collaborators (1998) reported that optimal discoloration was closely related to optimal growth and that overall discoloration was obtained in the pH range of five to eight, while at extreme pH levels, an increase in color was observed [42].

With respect to the color of DSW treated with *A. niger*, *F. flavus*, *T. versicolor* and *P. chrysosporium*, our results showed a lower impact, as compared to the literature. One of the most studied fungi for potential decolourization of distillery effluent was *Aspergillus sps*. *Aspergillus fumigatus* G-2-6, *Aspergillus niger*, *A. niveus*, *A. fumigatus* UB260 had an average of 55–79% decolourization [45,46,47,48,49,50]. Miranda et al. (1996) showed that, under optimal nutrient concentrations, aerobic treatment using *A. niger* allowed for a decolourization of beet molasses by 69%. Furthermore, they reported that 83% of the total color removed was eliminated biologically and 17% by adsorption on the mycelium [47]. Under optimal pH, Patil and collaborators (2003) showed that a melanoidin solution was decolourized from 60% to 72% by *A. niger* immobilized cells [51]. Raghukumar et al. (2001) reported that a diluted cane molasses stillage treated with *F. flavus* could reach up to 80% decolourization [43]. Further, aerobic treatment of a diluted molasses spent wash by *T. versicolor* had a decolourization yield of 53% [52]. When beet molasses were used, the decolourization yielded 58–81% OD_475 nm_ reduction. From 53.5 to 80% of decolourization of supplemented molasses spent wash treated by *P. chrysosporium* was reported [40,42]. Moreover, Fahy and coworkers (1997) demonstrated that the further addition of a carbon source like glucose in a 6.25% molasses spent wash medium strongly enhanced the decolourization yield from 49 to 80% by *P. chrysosporium* [44]. 

Some of our results were somewhat contradictory with other published works. For instance, a study showed that *C. tropicalis* could reach 75% decolourization level of a supplemented molasses spent wash when incubated at 45 °C [19]. Likewise, treatment of distillery spent wash with the ascomycetes of *Penicillium* genus resulted in about 50% reduction of the color [46]. With reference to *Thanatephorus cucumeris* (Rhizoctonia sp. D-90), Sirianuntapiboon and coworkers (1995) reported the decolourization of a melanoidin medium (molasses) by 87.5% thanks to an absorption mechanism. Indeed, the pigments were accumulated in cytoplasm and around the cell membrane before their degradation by intracellular enzymes [53]. To the best of our knowledge, no studies have focused on the decolourization of DSW by *Galactomyces geotrichum*, *Rhizopus microsporus*, *Giberella fujikuroi* and *Fusarium* sp. Notwithstanding this, considering their use for molasses decolourization, *Galactomyces geotrichum* and *Rhizopus microsporus var. oligosporus* could achieve a color reduction of diluted molasses of up to 87% and 38%, respectively [36]. Similarly, Seyis and Subasioglu (2009) showed that molasses decolourization by *Gibberella fujikuroi and Fusarium* species were not successful [54]. The OD_475 nm_ increase could result from pigments repolymerization, from a higher rate of nutriment consumption and from production by the microorganism of molecules that also absorb at this wavelength [55,56,57].

### 3.3. Effect of Aerobic Treatment on pH

Compared to the initial pH of the DSW broth (in the range of 4.77–4.95), all microbial treatments of crude DSW led to a significant increase of final pH (Table 1). Alkalinisation of the medium may be the result of an ammonium release during the assimilation of nitrogen source like proteins for the microorganism growth or a consumption of organic acids or reducing sugar present in DSW [55]. Among the 37 strains tested in this study, 22 could achieve a pH final value above 8 units. Among the best alkalinising strains, maximum pH (>9 units) was reached for DSW incubated with *A. terreus var. africanus* (9.05), *P. verrucosum* (9.03) and *A. terreus var. terreus* (9.0). More generally, among the *Aspergillus* and anamorphs genera, seven strains were found to reach a pH of above 8.3 units. 

Several studies have shown that the degradation of melanoidins, which is related to discoloration, tends to increase with alkaline pH. For instance, Hayase and collaborators (1984) reported that the discoloration of melanoidin occurred more rapidly at alkaline pH than at acidic or neutral pH and could reach up to 94% discoloration at pH 10 [58]. In addition, Mohana and coworkers (2007) reported that melanoidins are less soluble in acidic rather than in alkaline pHs and that pHs less than or greater than 7 units lead to a decrease of discoloration activity [59]. Similarly, Agarwal and collaborators (2010) claimed that melanoidins were more soluble at alkaline pH [60]. 

Contrary to these studies, we found no specific link between pH and (dis)colorisation of DSW was shown (see Table 1). Indeed, DSW aerobic fermentations using *A. terreus var. terreus* and *Penicillium verrucosum* led, in both cases, to an alkalinisation of the supernatant pH of DSW up to 9 units, but in the first case, an OD_475 nm_ decrease of 38.84% could be noticed, whereas an OD_475 nm_ increase of 68.99% was observed in the second case. Likewise, *A. oryzae* and *F. flavus* induced a decolourization of DSW by about 22%, but an alkalinisation of pH of 8.86 and 6.17, respectively.

As few sugar remain in residues like sugarcane molasses after sugar fabrication, the ethanol production from these residues conduced the use of harsher processing steps to depolymerize the structural polysaccharides. These processes result in side reaction products and in the acidification of the medium that are potentially inhibitory to microbial growth. Therefore, anaerobic digestion of the vinasse produced from sugarcane molasses may be fraught with problems [61]. As aerobic fermentation of DSW by yeasts and filamentous fungi bring about alkalinisation of DSW, the anaerobic digestion of the latter could be improved.

### 3.4. Biomass Production and Mineral Content of DSW after Aerobic Treatment 

The biomass production of the 37 yeasts and filamentous fungi strains was measured during growth on crude DSW (Table 1). Microorganisms that presented the best production of biomass during aerobic treatment of DSW were *Trametes hirsuta* (29.40 g·L^−1^), *A. terreus var. africanus* (29.19 g·L^−1^), *Clavispora lusitaniae* (28.56 g·L^−1^), *T. versicolor* (25.96 g·L^−1^) and *Issatchenkia orientalis* (25.41 g·L^−1^). In the same way as COD, OD_475nm_ and pH, we again found that the *Aspergillus* genus was particularly efficient in biomass production on crude DSW. The 9 *Aspergillus* anamorphs strains showed that biomass productions, after 10 days incubation, were comprised of between 17.98 g·L^−1^ (*Fennellia flavipes*) and 29.19 g·L^−1^ (*Aspergillus terreus var africanus*). Smaller amounts of biomass were observed for aerobic fermentation of crude DSW by the yeasts such as *P. jadinii* and *S. cerevisiae* (14.67 and 11.67 g·L^−1^, respectively).

Several studies have concluded that the COD reduction and/or decolourisation of diluted and/or supplemented molasses spent wash from sugarcane or sugar beet feedstocks by strains of *Aspergillus*, *Penicillium*, *Candida* and *Pichia* genus was accompanied by a fungal growth on the medium [62]. Biomass productions in DSW treated by *Aspergillus* and anamorphs strains were somewhat higher than those previously reported in literature. For instance, Rosalem and collaborators (1985) showed that biomass production of *Aspergillus niger* grown on DSW could vary from 8 to 13 g·L^−1^ [32]. Likewise, cellular concentration of *Aspergillus oryzae* grown on DSW were comprised between 12 and 17 g·L^−1^ dry weight [31,33]. In their study, Rolz and collaborators (1975) also demonstrated that biomass production by *Penicillium* sp. grown on DSW can reach up to 16 g·L^−1^ [30]. 

Data from the literature showed that *Issatchenkia orientalis* incubated in DSW supplemented with molasses, MgSO_4_, urea and H_3_PO_4_ could only produce a biomass of up to 8 g·L^−1^ [63]. The growth of *S. cerevisiae* on molasses stillage reached a maximum biomass production of about 12.7 g·L^−1^ [64]. Similarly, growth of *P. jadinii* on DSW supplemented on molasses produced from 9 to 18 g·L^−1^ of dry biomass [65]. Our results therefore clearly indicate that aerobic treatment of crude DSW by these filamentous fungi and yeast strains could achieve a significant reduction of polluting loads of DSW concomitantly with a high production of dry biomass (Table 1) that could be further valuated into added value molecules. Unexpectedly, our study did not reveal a clear link between biomass production and COD reduction (Table 1). This was particularly true for the strains that grow poorly on DSW (biomass production of *P. antarctica*, *P. rugulosum*, *P. angusta* and *G. fujikuroi* were comprised between 0.75 and 4.12 g·L^−1^), but showed a significant decrease in COD ranging from 38% to 62%. This result indicated that the enzymatic process of the reduction of polluting loads could work independently of the process of using nutriments from DSW for growth. 

We also noticed that aerobic treatment by the 37 strains used in this study always resulted in a significant reduction of mineral content of DSW (Table 1). This decrease was considerable after treatment of DSW by *F. flavus* (61.5%), *A. terreus var. africanus* (66%), *A. oryzae* (66.6%), *P. chysosporium* (70.5%), *A. terreus var. terreus* (72.4%), *A. alutaceus* (73.5%) and *A. niger* (77.6%). In agreement with our results for COD, OD_475 nm_, pH and biomass production, we found that seven out of the nine *Aspergillus* and anamorphs strains showed a mineral reduction in the broth by at least 50%. This result confirmed the high potential of *Aspergillus* genus to efficiently reduce the polluting load of DSW concomitantly with a high valuable biomass production. Aerobic treatment conducted with *C. tropicalis* (20.8%), *P. angusta* (20.8%), *A. flavus* (20.9%), *P. antarctica* (22.1%), *C. glabrata* (26.6%), *P. guilliermondii* (28.2%), *C. lusitanea* (28.4%) and *C. albicans* (29.3%) led to a lesser, but significant decrease in mineral content. The growth of microorganisms is strongly dependent on micronutrients (such as iron, copper, manganese, zinc, and nickel) and macronutrients (like potassium, phosphorus, magnesium, nitrogen, sulphur, and calcium). These nutrients are involved in carbohydrate metabolism, amino-acids and vitamins production, Krebs cycle, nucleic acid production, pigments production and enzyme activities [66,67]. However, the absence of clear relationship between mineral content and biomass production may suggest that other phenomena are involved in the reduction of minerals in the media. For example, mineral content may decrease from precipitation as a consequence of DSW alkalinisation during aerobic treatment. 

### 3.5. Statistical Relationships between Physico-Chemical Parameters

A Principal Component Analysis (PCA) was carried out to group the strains according to their performances on the physico-chemical parameters of DSW (biomass production and variations of pH, minerals content, COD and OD_475nm_) and we investigated possible correlations between some of them. The Pearson correlation matrix showed that variables were moderately correlated between them (Table 2). 

**Figure 1:**
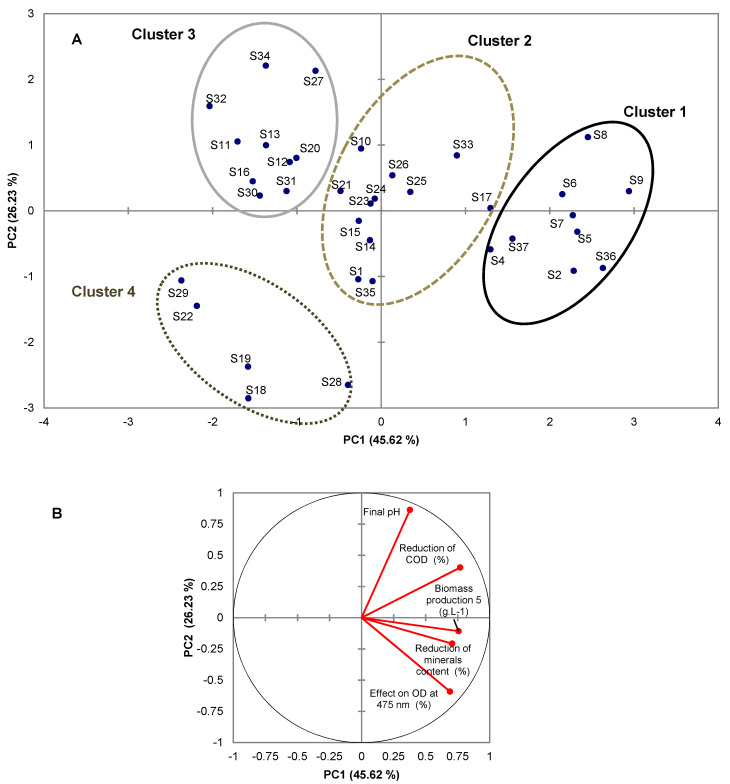
Score plot of PC_2_ versus PC_1_ (**A**,**B**) for fungal ability for DSW bioremediation.

For instance, we detected some correlations for pH and COD reduction (with a Pearson correlation coefficient *r* of 0.508), reduction of minerals content and effect on OD_475 nm_ (*r* = 0.503), biomass production with COD reduction on the one hand (*r* = 0.466) and the effect on OD_475 nm_ on the other hand (*r* = 0.447). Applied to the five original variables, the Cattell’s scree diagram [68] highlighted three significant Principal Components (PC) explaining 84.89% of the total variance, 45.62% for PC_1_, 26.23% for PC_2_ and 13.81% for PC_3_ (Appendix A—Table A1). The active coordinates retained by PCA were used to create Figure 1A,B. 

Principal Component Analysis was performed using XLSTAT (Addinsoft). Predicted groups were correlated to CAH clusters. Cluster 1, consisted in strains S2, S3, S4, S5, S6, S7, S8, S9, S36 and S37 which had the most significant COD decreases and biomass production. Cluster 2 includes strains S1, S10, S14, S15, S17, S21, S23, S24, S25, S26, S33 and S35 that had a COD decrease and biomass production yields less higher than ones of the strains of cluster 1. Cluster 3, consisted in strains S11, S12, S13, S16, S20, S27, S30, S31, S32 and S34, which significantly increase OD_475 nm_. The remaining strains (S18, S19, S22, S28 and S29) constituted the last group (Cluster 4) and had a less important effect on the final pH.

The eigenvectors of the covariance calculated enabled the defining of three PCs (Table 3). Only the original variables, whose correlation values with the principal components were greater in absolute value than 0.5, were taken into account. The first axis PC_1_ was representative of a global average level of the variables and strongly correlated with four of the five parameters (Appendix A—Table A2). These four parameters (COD reduction, biomass production, minerals content reduction and effect on OD_475 nm_) contributed for 93.77% to PC_1_ construction. Additionally, the variables (final pH and the effect on the OD value at 475 nm) are in absolute value the original variables best correlated with the PC2 axis. PC_2_ axis (Appendix A—Table A2). It can be noted that PC_2_ was mainly built by the pH and the effect on OD_475 nm_ variables, i.e., 83.53% of contribution to PC_2_ construction. Surprisingly, we found that some fungal species, such as *P. antarctica* (S32), had very little growth on DSW (0.75 g·L^−1^) despite a high COD consumption and a significant increase of pH, while species like *P. chrysosporium* (S28) showed significant biomass production (17 g·L^−1^), concomitant with small pH increase (7.01) and moderate COD consumption (23%). Then the third axis PC_3_ was built mainly on biomass production and minerals content reduction (86.56% of the PC_3_ construction). The variable reduction of mineral content also greatly contributed to the construction of the PC_3_ axis (Appendix A—Table A2). By opposition to *A. flavus* (S3), which turned out to produce a high amount of biomass (19.77 g·L^−1^), but a weak minerals consumption (20.88%), *P. rugulosum* (S26) could consume a large amount of mineral content (56.26%) with very little growth on DSW (2.36 g·L^−1^) *(*Figure 1A). These results suggested that a part of the minerals was indeed used for fungal growth, while another part was precipitated due to the alkalinisation of the DSW.

**Figure 2:**
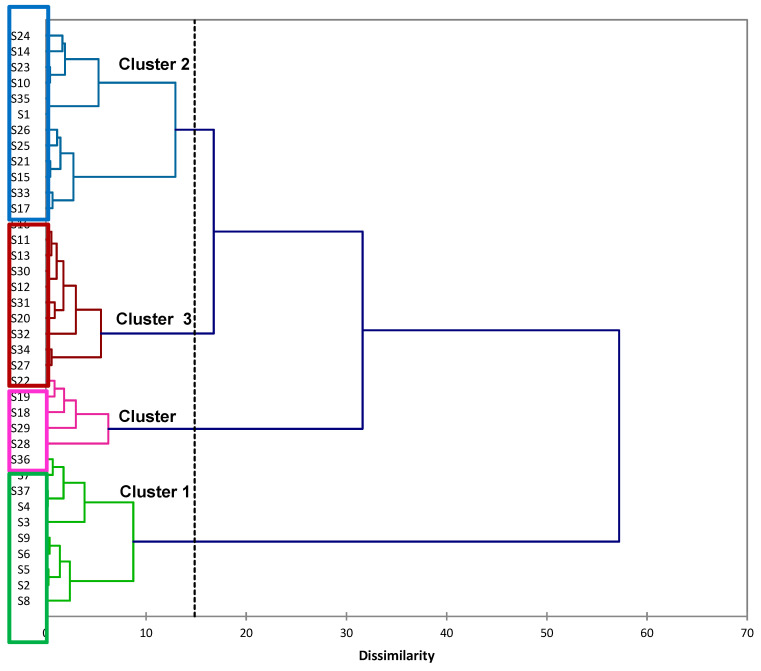
Hierarchical clustering of fungi species using Ward’s method and XLSTAT (Addinsoft).

PCA indicated that the strains could be classified into three to four groups. According to hierarchical cluster analysis (HCA), four groups of strains with close characteristics had been defined, explaining 64.62% of the total inter-variance and 35.38% of the total intra-variance (Figure 2). The distribution of the clusters according PC_1_ and PC_2_ (Figure 1A,B) allowed us to define the common characteristics of strains belonging to the same cluster (Appendix A—Table A3). Cluster 1 including the 8 *Aspergillus* anamorphs strains and the 2 *Trametes* spp. was characterized by aerobic treatment resulting in both high biomass production, high COD and mineral content reductions and a strong impact on OD_475 nm,_ resulting in significant decolourization. Cluster 3 included strains that led to a significant increase of OD_475 nm_ that could reach 190.9% in comparison to the OD_475 nm_ of crude DSW and conduced to the lesser mineral consumption. Cluster 4 consisted of strains whose effect on final pH was less important and that brought to a lesser biomass production and COD reduction. The final pH of DSW treated by strains defined in Clusters 1 and 3 were generally above pH = 8 whereas the pH of DSW treated by strains of Cluster 4 had a pH lower than seven. Cluster 2, which gathered all the other strains, was formed by strains that influenced COD and mineral contents and produced biomass on DSW, but less significantly than the strains of Cluster 1.

Automatic truncation based on entropy (dotted line) allowed identifying four consistent groups of fungi explaining 64.62% of the total inter-variance and 35.38% of the total intra-variance. Order of appearance of clusters (from top to down) was Cluster 2, Cluster 3, Cluster 4 and Cluster 1.

## 4. Conclusions 

Among the 37 strains studied, we demonstrated that species from the *Aspergillus and Trametes* genus generally gave the best results for bioremediation purposes with COD reductions reaching until 77%, decolourization yields until 43% and a significant alkalizing ability (pH increase of 4 units). While the data from the literature concerned diluted and/or supplemented vinasse, our study first reported the depolluting potential of these same strains on raw vinasse. Mitigation of the pollution potential of aerobically treated effluent compared to crude vinasse was reflected in the significant increase of pH and high COD and mineral consumptions. Utilization of filamentous fungi and yeasts for sugarcane vinasse treatment turned out to be very promising. Mixed cultures should be performed in order to improve the depollution yields. Moreover, some strains were able to show striking growth on raw DSW. Seven of the nine *Aspergillus* sp. strains, the white-rot fungi *Trametes versicolor* and *T. hirsuta* and two yeasts *Clavispora lusitanea* and *Issatchenkia orientalis* could reach biomass yields higher than 20 g·L^−1^. The high yields of fungal biomass produced (until 29 g·L^−1^) could constitute an easily recoverable substrate for the production of renewable energy through anaerobic digestion.

## Figures and Tables

**Table 1:** Strains used in this study, effects of aerobic treatment of DSW on physicochemical parameters and biomass production.

Strain Number	Strains (Genera/Specie)	MUCL Reference Number	Reduction of COD ^1^ (%)	Effect on OD at 475 nm ^2^ (%)	Reduction of Minerals Content ^3^ (%)	Final pH ^4^	Biomass Production ^5^ (g·L^−1^)
S1	*Arthroderma otae*	MUCL 41713	59.22	98.26	36.99	6.91	18.11
S2	*Aspergillus alutaceus*	MUCL 39539	69.23	58.12	73.49	7.91	21.7
S3	*Aspergillus flavus*	MUCL 19006	70.86	80.00	20.88	8.72	19.77
S4	*Aspergillus itaconicus*	MUCL 31306	73.23	64.64	40.94	7.64	21.41
S5	*Aspergillus niger*	MUCL 19001	70.11	73.04	77.57	8.31	21.25
S6	*Aspergillus oryzae*	MUCL 19009	65.98	77.97	66.62	8.86	24.35
S7	*Aspergillus parasiticus*	MUCL 14491	74.6	57.54	53.66	8.46	24.48
S8	*Aspergillus terreus var africanus*	MUCL 38960	76.53	110.14	66,00	9,00	29.19
S9	*Aspergillus terreus var terreus*	MUCL 38640	73.5	61.16	72.4	9.05	24.90
S10	*Candida albicans*	MUCL 30114	56.56	118.01	29.3	8.69	19.34
S11	*Candida dubliniensis*	MUCL 41201	45.98	152.53	32.18	8.45	10.29
S12	*Candida glabatra*	MUCL 29833	57.34	130.64	26.62	8.12	12.43
S13	*Candida tropicalis*	MUCL 29893	50.41	138.72	20.75	8.42	15.21
S14	*Clavispora lusitanea*	MUCL 29855	54.72	116.84	28.35	7.39	28.56
S15	*Colletotricum graminicola*	MUCL 44764	57.75	92.17	39.23	7.94	11.79
S16	*Cryptococcus albidus*	MUCL 30400	44.89	135.35	30.29	8.13	12.47
S17	*Fennellia flavipes*	MUCL 38811	58.65	75.65	61.46	8.74	17.98
S18	*Flavodon flavus*	MUCL 38427	28.99	77.10	37.48	6.17	14.98
S19	*Fusarium proliferatum*	MUCL 43482	34.44	91.59	53.83	6.37	6.38
S20	*Fusarium sporotrichioides*	MUCL 6133	55.13	140.00	43.81	8.25	8.6
S21	*Galactomyces geotrichum*	MUCL 43077	56.95	104.64	46.39	8.26	6.79
S22	*Gibberella fujikuroi*	MUCL 42883	37.89	106.96	36.58	6.76	4.12
S23	*Gibberella zeae*	MUCL 42841	55.8	109.28	33.52	8.04	20.9
S24	*Issatchenkia orientalis*	MUCL 29849	48.13	139.73	49.56	8.08	25.41
S25	*Komagatella pastoris*	MUCL 31260	69.7	107.41	56.84	7.99	9.1
S26	*Penicillium rugulosum*	MUCL 41583	62.48	86.38	56.28	8.72	2.36
S27	*Penicillium verrucosum*	MUCL 28674	62.09	168.99	49.05	9.03	8
S28	*Phanerochaete chrysosporium*	MUCL 38489	23.51	74.20	70.49	7.01	17
S29	*Pichia angusta*	MUCL 27761	49.52	114.14	20.78	6.53	4.04
S30	*Pichia guilliermondii*	MUCL 29837	54.78	138.38	28.21	7.54	12.23
S31	*Pichia jadinii*	MUCL 30058	40.91	141.08	46.33	8.21	14.67
S32	*Pseudozyma antarctica*	MUCL 47637	51.33	136.03	22.11	8.9	0.75
S33	*Rhizopus microsporus var oligosporus*	MUCL 31005	67.32	95.94	52.04	8.85	14.66
S34	*Saccharomyces cerevisiae*	MUCL 39449	55.84	190.91	41.17	8.88	11.64
S35	*Thanatephorus cucumeris*	MUCL 43254	65.28	105.22	44.63	6.66	16.32
S36	*Trametes hirsuta*	MUCL 40169	74.01	57.54	62.86	7.8	29.4
S37	*Trametes versicolor*	MUCL 44890	73.64	67.54	39.05	7.79	25.96

^1^ Reduction of COD corresponded to ratio between COD removal during aerobic fermentation and initial COD of crude DSW based upon 100%; ^2^ Evolution of OD measured at 475 nm was the relation between OD after aerobic fermentation and OD of crude DSW based upon 100%; ^3^ Reduction of minerals content corresponded to ratio between minerals removal during aerobic fermentation and initial minerals content of crude DSW based upon 100%; ^4^ Final pH measured in DSW broth after 10 days of fermentation; ^5^ Biomass production was determined according to Materials and Methods (Section 2.1 and Section 2.2) and corresponded to the difference between biomass obtained after DSW filtration at the end of 10 days fermentation and biomass inoculated on DSW at the beginning of the fermentation. Biomass inoculated was the sum between fungal cells obtained after growth on MA broth and total solids content naturally contained on DSW.

**Table 2:** Pearson correlation matrix.

Parameters	Reduction of COD (%)	Effect on OD at 475 nm (%)	Reduction of Minerals Content (%)	Final pH	Biomass Production (g·L^−1^)
**Reduction of COD (%)**	**1**	**0.344**	0.289	**0.508**	**0.466**
**Effect on OD at 475 nm (%)**	**0.344**	**1**	**0.503**	−0.197	**0.447**
**Reduction of minerals content (%)**	0.289	**0.503**	**1**	0.179	**0.355**
**Final pH**	**0.508**	−0.197	0.179	**1**	0.135
**Biomass production (g·L^−1^)**	**0.466**	**0.447**	**0.355**	0.135	**1**

Bold values are significantly different from 0 at a significance level α = 0.05.

**Table 3:** Correlations between the parameters and principal components.

Parameters	PC1	PC2	PC3
Reduction of COD (%)	0.769	0.402	−0.234
Effect on OD at 475 nm (%)	0.690	−0.591	−0.017
Reduction of minerals content (%)	0.707	−0.207	0.637
Final pH	0.377	0.864	0.195
Biomass production (g·L^−1^)	0.756	−0.107	−0.439

## Appendix A

**Table A1:** Eigenvalues obtained from PCA.

	PC_1_	PC_2_	PC_3_	PC_4_	PC_5_
**Eigen value**	2.28	1.31	0.69	0.49	0.23
**Variability (%)**	45.62	26.23	13.81	9.72	4.61
**Cumulative variability (%)**	45.62	71.85	85.66	95.39	100.00

**Table A2:** Eigenvectors obtained from PCA.

Parameters	PC_1_	PC_2_	PC_3_
Reduction of COD (%)	0.509	0.351	−0.281
Effect on OD at 475 nm (%)	0.457	−0.516	−0.021
Reduction of minerals content (%)	0.468	−0.181	0.766
Final pH	0.250	0.754	0.234
Biomass production (g·L^−1^)	0.501	−0.094	−0.528

**Table A3:** PCA and HCA data related to the five original parameters.

		Biomass Production (g·L^−1^)	Reduction of COD (%)	Reduction of Minerals Content (%)	Final pH	Effect on OD at 475 nm
**All data**	Min.	0.75	23.51	20.75	6.17	190.91
	Max.	29.4	76.53	77.57	9.05	57.94
	Aver.	15.85	57.5	45.07	8.02	105.24
	S.D.	7.69	13.24	16.25	0.81	33.39
**Cluster 1**	Min.	19.77	65.98	39.05	7.79	110.14
	Max.	29.4	76.53	77.57	9.05	42.46
	Aver.	24.24	72.17	57.35	8.35	70.77
	S.D.	3.3	3.1	18.36	0.54	16.13
**Cluster 2**	Min.	2.36	48.13	28.35	6.66	139.73
	Max.	28.56	69.7	61.46	8.74	75.65
	Aver.	15.94	59.38	44.55	8.02	104.13
	S.D.	7.55	5.97	11.12	0.72	16.6
**Cluster 3**	Min.	0.75	40.91	20.75	7.54	190.91
	Max.	15.21	62.09	49.05	9.03	130.64
	Aver.	10.63	51.87	34.05	8.39	147.26
	S.D.	4.17	6.46	10.26	0.45	18.83
**Cluster 4**	Min.	4.04	23.51	20.78	6.17	114.14
	Max.	17	49.52	70.49	7.01	74.2
	Aver.	9.30	34.87	43.83	6.57	92.8
	S.D.	6.22	9.84	18.94	0.33	17.68
